# A mobile app to challenge obsessional beliefs in adolescents: a protocol of a two-armed, parallel randomized controlled trial

**DOI:** 10.1186/s12888-024-05735-x

**Published:** 2024-04-09

**Authors:** Yuliya Saman, Belén Pascual-Vera, Marta Corberán, Sandra Arnáez, María Roncero, Gemma García-Soriano

**Affiliations:** 1 Departamento de Personalidad, Evaluación y Tratamientos Psicológicos, Universitat de València, Avda. Blasco Ibáñez, 21, Valencia, 46010 Spain; 2 Departamento de Personalidad, Evaluación y Tratamientos Psicológicos, Universidad Nacional de Educación a Distancia, C/ Bravo Murillo, 38, Madrid, 28015 Spain

**Keywords:** Obsessive-compulsive disorder, Maladaptive beliefs, mHealth application, Adolescence, Protocol, Randomized controlled trial

## Abstract

**Background:**

Adolescence is a crucial stage for the development of OCD symptoms that, in most cases, persist into adulthood. This requires designing preventive strategies tailored to this population. Therefore, we aim to describe the study protocol that will be used to examine the effectiveness of a mobile health application to challenge obsessional beliefs in adolescents.

**Methods:**

A two-armed randomized controlled trial will be conducted on an adolescent sample from the general population. The experimental group will use the intervention module (GGOC-AD) of a mobile app on the GGtude platform for 14 days whereas the control group will use a non-active module (GGN-AD) of said app. Primary outcome measures will be obsessional beliefs and obsessive-compulsive symptoms, and secondary measures will be self-esteem and emotional symptoms. Three assessment points will be conducted at baseline, post-intervention, and one-month follow-up. A linear multiple regression model with an intention to treat approach will be used. The expected total sample size will be 55 participants.

**Discussion:**

We expect that the intervention group will show a reduction in obsessional beliefs and OCD-symptoms at post and follow-up in comparison with the control group. Additionally, we expect that the app will improve participants’ self-esteem. This study could provide an accessible mobile health tool to prevent OCD-related symptoms in adolescents.

**Trial registration:**

ClinicalTrials.gov identifier: NCT06033391. Registered September 4, 2023.

**Graphical Abstract:**

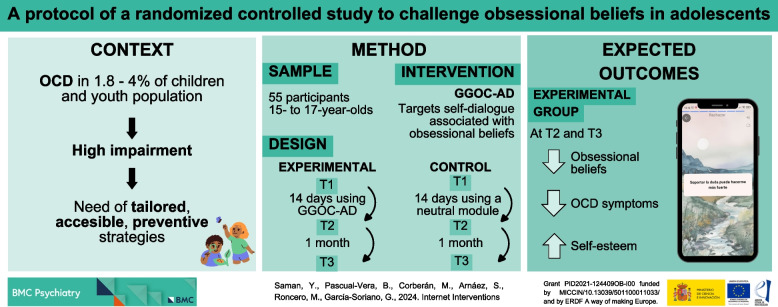

**Supplementary Information:**

The online version contains supplementary material available at 10.1186/s12888-024-05735-x.

## Background

Obsessive-compulsive disorder (OCD) is a highly distressing disorder that affects between 1.8–4% of the child and youth population and up to 5.5% of children have subclinical OCD symptoms [1, 2]. Studies indicate that OCD often begins during childhood or adolescence in 57% of patients and persists into adulthood in approximately 40% of cases [3, 4]. Left untreated, OCD is associated with substantial impairment in academic and psychosocial functioning, family-related difficulties, and diminished quality of life [5, 6]. Moreover, the early onset of OCD and its longer duration have been linked to OCD persistence [7].

Cognitive-behavioral therapy (CBT) has been extensively supported as the “gold standard” for treating OCD. A stepped-care approach [8] recommends CBT for children and young individuals with OCD, specifically for those experiencing moderate to severe functional impairment and for those with mild OCD who have not benefited from or declined guided self-help. Despite the effectiveness of CBT, accessing face-to-face treatment is not always easy due to a delay in help-seeking, limited availability of trained professionals, and treatment costs [9–11]. To overcome treatment barriers, mobile health (mHealth) platforms offering CBT-based interventions have been proposed [12, 13]. These platforms provide continuous accessibility via personal devices, are familiar and user-friendly for young people, and are cost-effective. Indeed, such interventions are coherent with the stepped-care approach for OCD [8], which attempts to provide the most effective but least intrusive treatment appropriate for a person’s needs and difficulties. 

Despite the fact that the implementation of mHealth applications in OCD is brief [14], there is notable evidence suggesting the potential benefits of CBT-based mobile apps for this disorder. One such mHealth app in OCD is GGtude (Herzliya, Israel), a mobile application platform with various modules that have shown to be effective in reducing maladaptive beliefs and symptoms related to various psychological difficulties [15–18]. Two of these modules are based on cognitive-behavioral theories of OCD and designed to challenge maladaptive beliefs that underlie common OCD symptoms (OC) (e.g., contamination) as well as relationship obsessions (RO). Cognitive-behavioral theories propose that maladaptive beliefs and misinterpretations associated with the occurrence of common unwanted intrusive thoughts lead individuals to engage in a variety of counterproductive behaviors. Consequently, a key target in OCD treatment is to decrease symptoms by challenging maladaptive beliefs and related dysfunctional behaviors [19, 20].

Several studies have supported the effectiveness of the modules of the GGtude platform in reducing obsessional beliefs. In general, for the adult population, Roncero et al. [18, 21] showed that daily cognitive training using the GGtude platform for OCD (OC and RO modules) decreases OCD-related maladaptive beliefs. Similarly, Cerea et al. [22] and Akin-Sari et al. [23], replicated these results in participants with subclinical levels of relationship-related-OCD symptoms and OCD symptoms, respectively. Findings also suggest that challenging maladaptive beliefs impacts OCD symptoms. For instance, Roncero et al. [18], reported a significant pre-post training decrease in the levels of OCD symptoms and showed that the changes observed in OCD-related maladaptive beliefs were associated with a reduction in OCD symptom levels. Similarly, using cross-over designs, Roncero et al. [21] and Cerea et al. [22] also observed reductions in OCD symptoms (using the Obsessive-Compulsive Inventory – Reduced; OCI-R) after the use of the app. In the same vein, Akin-Sari et al. [23] reported a large reduction in the OCI-R scores of participants at risk of OCD, which could be attributable to the effect of the app, suggesting that the OC module could be a promising tool for early intervention in OCD. Finally, Pascual-Vera et al. [24], provide evidence in favor of these assumptions in clinical samples. In a single case study, the OC module was used as a prevention relapse component of a CBT face-to-face treatment. Findings show that after the use of the OC module, the effects of CBT treatment were maintained and scores in OCD symptoms and OCD-related maladaptive beliefs improved.

Building upon the promising findings of the GGtude platform for OCD in adult samples, we have developed an adaptation of the OC module for children and adolescents, which we have named GGOC-AD (Obsessive-Compulsive Disorder – Adolescents). Adolescence, which is a critical transitional period where individuals shape their identity, build relationships with peers, and develop their academic studies, underscores the need to design specific strategies tailored to this population [25, 26]. By extending the OC module to adolescents, we hope to contribute to the prevention of OCD by offering a novel, user-friendly, and accessible app focused on challenging maladaptive beliefs relevant to OCD. The objective of this study will be to describe the study trial used to examine the efficacy of the GGOC-AD module on 15- to 17-year-old adolescents. Specifically, we will analyze the impact of the module on OCD-related maladaptive beliefs and obsessive-compulsive symptoms. Secondarily, we will explore its effects on self-esteem and emotional symptoms.

## Methods

### Study design and procedure

A two-armed, parallel-design randomized controlled superiority trial will be conducted to evaluate the effectiveness of the GGOC-AD module intervention for adolescents compared to a non-active module (GGN-AD). The randomization procedure is described in detail in the subsection 2.5. The present study protocol complies with the Standard Protocol Items: Recommendations for Interventional Trials (SPIRIT) checklist.

**Fig. 1:**
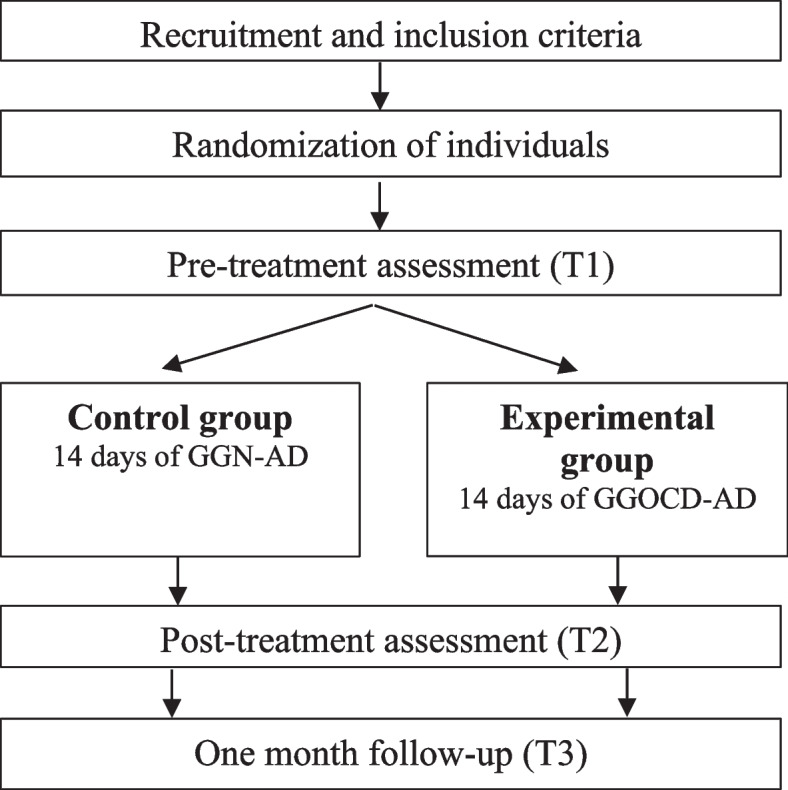
Flow diagram of the study design *Note.* 14 days of GGN-AD: use of the GG Neutral – Adolescents module during 14 days; 14 days of GGOC-AD: use of the GG Obsessive-Compulsive Disorder – Adolescents module during 14 days

Figure 1 depicts the trial design and times of assessment. Students will be recruited from public schools in the metropolitan area of Valencia (Spain). Researchers will invite different schools to participate in the study. After the education center agrees to participate, members of the research group will meet students in the designated classroom, provide information about the study, and invite them to participate. Students will then receive a sheet with information about the study and the informed consent for parents or guardians and the student. Finally, participants that explicitly agree to participate, provide the informed written consent, and meet the inclusion criteria will be randomized in two groups: the intervention group that will use GGOC-AD and the control group that will use the GGN-AD module. On weekdays, students will complete the app during school hours at a pre-arranged time with the education center in order to interfere as little as possible. At least two members of the research group will be available to assist in the assessment sessions (T1-T3) and monitor the use of the app in order to promote participants’ retention. Students will be asked to complete the app at home during the weekend. As shown in Fig. 1, on the first day of the study, participants will be requested to complete the baseline assessment measures (T1, pre-treatment) and download the GGtude platform app using their personal mobile device. It will be freely available from the App Store or Google Play. The intervention group will access the GGOC-AD module of said app and the control group will use the GGN-AD module. Users will carry out cognitive training for 14 consecutive days, completing approximately three levels per day. On the day that they finish the app (day 14), participants will be instructed to complete the assessment instruments again (T2, post-treatment). Finally, a one-month follow-up evaluation will be conducted (T3). Both groups will complete the outcome measures and the app at about the same time, using their personal mobile device. At all-time assessments, questionnaires will be completed by Lime Survey software in a designated classroom.

### Participants

This study will be conducted in a formal educational setting with students from schools in the metropolitan area of Valencia (Spain). The expected age of participants will be between 15 and 17 years old. Inclusion criteria for participants will be: a) providing written, informed consent (both the adolescent and their parents or legal guardian), b) being enrolled in 4th level of Obligatory Secondary Education of the Spanish Education System (ISCED levels 2 and 3 in the International Standard Classification of Education), and c) availability to use a mobile phone (indistinctly Android or iOS mobile system) with Internet access.

### Intervention

Both intervention and control modules will be administered in the school center. Participants will use the module on their own mobile device individually. During weekdays, they will use it in their classroom with the assistance of researchers, whereas on weekends they will be required to complete it at home on their own.

#### GG obsessive-compulsive disorder – adolescents (GGOC-AD) module

The intervention module will be GGOC-AD. It consists of game-like interactions that are intended to help users increase their accessibility to self-statements that facilitate more adaptive appraisals of thoughts, emotions, and coping strategies. Users of GGOC-AD will be presented with “blocks” featuring statements related to OCD relevant beliefs, such as “I can’t trust my memory” or “No one is perfect”. Participants will be instructed to respond to these statements by either accepting them (i.e., pulling the blocks downward toward themselves) or rejecting them (i.e., throwing the blocks upward away from themselves). If a participant makes a wrong choice, that is, accepts a statement representing an OCD-related maladaptive belief, a notification will appear encouraging them to try again. GGOC-AD is composed of a total of 57 levels that are divided in 14 blocks. Users are instructed to complete one block, that is, three to four levels per day. Each level has approximately eight to 10 statements, which take approximately 1 minute to complete.

The GGOC-AD module was designed based on the Spanish version of the OC module previously used in the non-clinical adult population and OCD patients [18, 21, 24]. The adolescence adaptation was carried out as follows: first, the 968 statements that compose the adult version were reviewed, one by one, by independent members of the research group. Each reviewer selected the ones that could be problematic for adolescents, considering: a) the lack of compressibility of the vocabulary and b) the contextual inappropriateness for teenagers. For instance, sentences that referred to a working environment or to a romantic relationship were considered problematic due to being difficult to generalize to the adolescent population. A total of 370 statements were proposed to be reviewed. To that end, three separated blocks of statements were administered to a group of nine adolescents from different schools. The adolescents were instructed to carefully read each statement and evaluate each one according to a 10-point Likert scale (from 1 = “I don’t understand it at all” to 10 = “I understand it completely”). After that, they had to propose a better alternative for those that were difficult to understand (scores < 8). The research group discussed each of the sentences and the alternatives proposed by the adolescents in different sessions and agreed to change a total of 75 statements by consensus. With this version of the OC module, five adolescents completed the module over 14 days. After using the module, the research group members met the students individually to ask for their opinion on the statements. Considering the feedback given by the participants, the research group reviewed all the statements in the module again. Finally, a total of 335 statements (34.6%) were adapted (e.g., “My partner accepts me” was changed to “My friends accept me”).

#### GG neutral – adolescents (GGN-AD) module

The control module will be GGN-AD. It is a non-active intervention that follows a similar structure and functioning to the intervention module. It includes the same number of levels as GGOC-AD and participants will also complete three levels per day. The main difference from the intervention module is the statement content. In this case, the statements are true/false options related to general knowledge, geography, math, or language issues. Participants will then have to accept the statements that are *true*, dragging them to the bottom of the screen (e.g., “Madrid is the capital of Spain”); and reject *false* sentences, dragging them to the top of the screen (e.g., “China is the capital of Spain”). The sentences of GGN-AD were reviewed and adapted to the Spanish context by the research team, because they were originally written to suit the English-speaking population. For example, phrases such as “Arizona is a state of the USA” were changed to “Valencia is a Spanish city”.

### Primary and secondary outcome measures

All measures will be completed at pre-, post- and follow-up assessment. Participants will also complete a sociodemographic data sheet (*see additional measures*). The primary outcomes measures will be:

- *Obsessive Compulsive Inventory - Child Version* (OCI-R-CV) [27]. The OCI-R-CV is a self-report instrument that assesses the discomfort associated with obsessive-compulsive symptoms in children and adolescents between 8 and 18 years old. It consists of 21 items with a 3-point Likert scale (from 0 = “never” to 2 = “always”). The total score and the six subscales (Doubting, Obsession, Hoarding, Washing/Checking, Ordering, and Neutralizing) will be used in this study. The Spanish validation of the OCI-R-CV shows good psychometric properties [28].
- *Obsessive Beliefs Questionnaire - Child Version* (OBQ-CV) [29]. The OBQ-CV is a self-report questionnaire that assesses obsessive beliefs through 44 items with a 5-point Likert scale (from 0 = “strongly disagree” to 4 = “strongly agree”). In this study, we will consider the three subscales: a) Responsibility and threat estimation, b) Perfectionism and intolerance of uncertainty, and c) Importance and need to control thoughts, and the OBQ-CV total score. The Spanish adaptation presents good psychometric properties [30].

The secondary outcome measures will be as follows:

- *The Patient Health Questionnaire for Depression and Anxiety* (PHQ-4) [31, 32]. This is a brief-screening to assess anxiety and depression symptoms. It is composed of 4 items evaluated with a 4-point Likert scale (from 0 = “never” to 3 = “almost every day”). The total score and the two subscales (anxiety and depression) will be used in this study. The Spanish validation shows good psychometric properties [33].
- *Single-Item Self-Esteem Scale* (SISE) [34]. The SISE is a self-report measure that determines the extent to which the sentence “I have a high self-esteem” describes participants on a 9-point scale, ranging from 1 (“not very true for me”) to 9 (“very true for me”). In this study, the SISE will be used as a self-esteem score, in accordance with the reliability and criterion validity reported by Robins et al. [34].

Additional measures:

- *Sociodemographic data sheet:* The data required will be the following: age, gender, data about their school center (academic year, class group, and school center).

### Randomization

All the participants will be randomly assigned to GGOC-AD (intervention) or GGN-AD (control) groups (allocation 1:1) with the randomizer.org platform (no block randomization). It will take place prior to the T1 assessment and will be conducted by a researcher not involved in the assessment and intervention procedure.

### Data management

Data will be collected and managed in line with the Ethics Committee of Research in Humans of Universitat de Valènica. Participants will access the questionnaires using a unique personal identification number (ID), created beforehand. It is a code that ensures anonymity and does not allow participants to be identified. Their ID will be used to match the collected data. No other personal data is requested. Data from the questionnaires will be stored on a secure server of the Universitat de Valènica. In addition, persons who do not adequately answer the two control questions (e.g., “In this question, please check “always”) included in the questionnaires at each evaluation time will be eliminated from the data base.

### Statistical analyses and sample size

Analyses will be performed using the free software R. The effect of the intervention on primary and secondary measures over time will be appraised using a generalised linear mixed model (GLMM), and, specifically, a linear multiple regression model where time (T1, T2 and T3) and group (experimental and control) are the independent variables whereas the primary and secondary outcome measures are the dependant variables. To avoid overly optimistic estimates of the effectiveness of the experiment, an ITT (intention to treat) approach will be used, using the method of multivariate imputation by chained equations algorithm (MICE). Descriptive statistics such as means, standard deviations, and frequencies will be also calculated.

The sample size of the study has been estimated a priori using G-power 3 [35] based on the formula provided by Cohen [36]. It was revealed that a sample of at least 55 participants will need to be recruited to accomplish a power study of .805 at alpha = .05 and a medium size effect (.15). Considering the dropout rates in previous studies in our context [21], we will invite approximately 200 individuals to participate in the current study.

## Discussion

This study aims to describe the protocol for a randomized control trial of a tailored module of a mobile app to challenge OCD-related maladaptive beliefs in adolescents. It responds to the need to design preventive strategies in OCD, with children and adolescents being a target population due to the prevalence rates of OCD and the history of subclinical obsessive-compulsive symptoms that often precede the full-blown disorder. The rationale and design of the GGOC-AD module focuses on maladaptive beliefs relevant in OCD, proposed as a cognitive vulnerability factor, that could lead to OCD development [37, 38]. In a clinical sample of children and adolescents, Coles et al. [29] showed that OCD symptom severity and interference was related to obsessive beliefs and Wolters et al. [39] reported that the degree of adscription of these beliefs differentiated patients with OCD from community controls. Recently, Pozza et al. [37], who examined whether maladaptive beliefs longitudinally predict OCD, showed that several beliefs (e.g., perfectionism, intolerance of uncertainty, threat overestimation) predicted the later development of OCD symptoms. This suggests the need to specifically target these dimensions for early detection and preventive programs. Considering the effectiveness of GGtude modules for OCD in addressing maladaptive beliefs [18, 21–23], our main hypothesis is that following the use of the GGOD-AD module, participants will exhibit a reduction in OCD-related maladaptive beliefs. Furthermore, we expect that this change will also lead to a decrease in the distress associated with OCD symptoms, as previously reported by Akin-Sari et al. [23]. We expect no changes in those dimensions after using the GGN-AD module in the control group.

A secondary objective is to examine the effects of the GGOC-AD module on self-esteem. We hypothesize that participants who use the GGOC-AD module will show increased self-esteem levels following the intervention compared to their pre-intervention levels. We expect no changes in self-esteem after using the GGN-AD module in the control group. The emphasis on self-esteem is particularly relevant during adolescence, a critical period for identity development. Moreover, explanatory models of OCD have suggested the relevance of self-construction on OCD [40–42]. Indeed, Roncero et al. [21] reported improvements in self-esteem following the challenge of OCD-related maladaptive beliefs. In addition, we will evaluate the modules’ impact on emotional symptoms. This examination could help ascertain the module’s specificity in addressing OCD symptoms and related beliefs versus affective symptoms.

If the GGOC-AD module proves effective, it could be used as a preventive strategy for OCD. Firstly, the module could act as a protective mechanism against vulnerability factors of developing OCD, which also could have an indirect impact on its symptoms, constituting a universal prevention intervention. Moreover, the implementation of GGOC-AD in a formal educational context may have additional benefits. That is, the app is a user-friendly and accessible tool that could be easily administered in a developmentally natural environment. This would allow a large number of students to be involved regardless of their risk of OCD, as it is less stigmatizing for individuals and provides the opportunity to promote mental health in an educational setting. Finally, this initiative is intended not only to potentially prevent the escalation of OCD symptoms and for its early detection, but also to improve early intervention for this population, providing an accessible and cost-effective tool in accordance with a stepped-care approach for OCD treatment (NICE, 2005). In fact, research on a case study with an adult OCD patient suggests its effectiveness as a relapse prevention tool [24].

Several limitations of our study are also expected. One difficulty could be adherence to the use of the app, due to the intervention context and the age range of participants. We will try to overcome this limitation by assisting students in the educational setting during the implementation of this study. Members from the research group will attend the classrooms every day to monitor students and help to make it familiar and natural for participants. Additionally, we are aware that the academic context might influence the study results. Thus, we plan to start the study and conduct all the assessment sessions in a period with the same level of academic requirements, preferably low.

Despite the limitations, we hope that this study could help to improve the well-being and mental health of children and adolescents. The implementation of a tailored mental health app for adolescents could contribute to the prevention of OCD. Future studies are needed to identify early signs of OCD and vulnerability risk factors for the early implementation of effective preventive strategies.

### Supplementary Information

**Supplementary Material 1.**

## Data Availability

Data availability or communication of the results is not applicable to this article as no new data were created or analyzed in the current study. However, the datasets generated/analyzed using this protocol will be anonymized and deposited in the Zenodo repository (https://zenodo.org/). Also, a report with the main results will be disseminated between participants and their parents through the educational center.

## Abbreviations

OCD: Obsessive-compulsive disorder
CBT: Cognitive-behavioral therapy
GGOC-AD: GG Obsessive-compulsive disorder – Adolescents
GGN-AD: GG Neutral – Adolescents
ID: Identification number
RO: Relationship obsessions
OC: Obsessive-compulsive
OCI-R-CV: Obsessive compulsive inventory - child version
OBQ-CV: Obsessive beliefs questionnaire - child version
PHQ-4: The patient health questionnaire for depression and anxiety
SISE: Single-item self-esteem scale
